# Association between PCSK9 Levels and Markers of Inflammation, Oxidative Stress, and Endothelial Dysfunction in a Population of Nondialysis Chronic Kidney Disease Patients

**DOI:** 10.1155/2021/6677012

**Published:** 2021-07-20

**Authors:** Evangelia Dounousi, Constantinos Tellis, Paraskevi Pavlakou, Anila Duni, Vasillios Liakopoulos, Patrick B. Mark, Aikaterini Papagianni, Alexandros D. Tselepis

**Affiliations:** ^1^Department of Nephrology, School of Health Sciences, University of Ioannina, Ioannina, Greece; ^2^Atherothrombosis Research Centre/Laboratory of Biochemistry, Department of Chemistry, University of Ioannina, Ioannina, Greece; ^3^Department of Nephrology, University Hospital of Patras, Patras, Greece; ^4^Division of Nephrology and Hypertension, 1st Department of Internal Medicine, AHEPA Hospital, School of Medicine, Aristotle University of Thessaloniki, Greece; ^5^Institute of Cardiovascular and Medical Sciences, University of Glasgow, Glasgow, UK; ^6^Department of Nephrology, School of Medicine, Aristotle University of Thessaloniki, Hippokration Hospital, Thessaloniki, Greece

## Abstract

Proprotein convertase subtilisin/kexin 9 (PCSK9) plays an important role in lipid metabolism while available literature regarding its involvement in the pathogenesis of atherosclerosis and in the expression of genes associated with apoptosis and inflammation is constantly increasing. Patients with chronic kidney disease (CKD) experience disproportionately increased cardiovascular morbidity and mortality due to dyslipidemia, accelerated atherosclerosis, inflammation, oxidative stress, and other risk factors. In the present cross-sectional study, we investigated the possible association of serum PCSK9 levels with markers of inflammation, oxidative stress, and endothelial damage in patients with CKD. *Patients and Methods*. Ninety-two patients with CKD stages II-*Ι*V (eGFR CKD-EPI 47.3 ± 25.7 ml/min/1.73 m^2^, mean age 66 years, 51 men) were included in the study. Plasma PCSK9 levels were correlated with comorbidities (arterial hypertension, diabetes mellitus, and history of cardiovascular disease), renal function indices (eGFR, proteinuria–UPR/24 h), lipid parameters (LDL-cholesterol, HDL-cholesterol, triglycerides, Lp(a), APO-A1, and APO-B), and soluble biomarkers of inflammation, oxidative stress, and endothelial damage (hs-CRP, fibrinogen, 8-epiPGF2a, ox-LDL, IL-6, TNF-*α*, sICAM-1, and sVCAM-1). *Results*. The mean plasma value of PCSK9 was 278.1 ng/ml. PCSK9 levels showed direct correlation with serum triglycerides (*p* = 0.03), Lp(a) (*p* = 0.01), and sICAM-1 levels (*p* = 0.03). There was no significant correlation between PCSK9 levels and indices of the renal function, other lipid profile parameters, inflammatory markers, or comorbidities. Multiple regression analysis showed a significant effect of Lp(a) on PCSK9 levels, and for each unit of higher Lp(a), an increase by 3.082 is expected (95% CI: 0.935-5.228, *p* = 0.006). At the same time, patients receiving statins are expected to have on average 63.8 ng/ml higher PCSK9 values compared to patients not receiving statins (95% CI: 14.6-113.5, *p* = 0.012). *Conclusion*. Plasma levels of PCSK9 in nondialysis CKD patients are correlated with endothelial dysfunction and lipid metabolism parameters. Statin intake increases PCSK9 levels significantly in this patient population. PCSK9 levels are not correlated with the severity of kidney disease. Major prospective studies are necessary to investigate the role of PCSK9 in the atherosclerotic cardiovascular outcome in CKD.

## 1. Introduction

Chronic kidney disease (CKD) has been characterized as an atherosclerosis multiplier increasing disproportionally the incidence of fatal and nonfatal cardiovascular events from the early stages [1]. Almost a quarter of patients with mild to moderate CKD have been reported to die mainly due to atherosclerotic cardiovascular disease (ASCVD) before the initiation of renal replacement therapy while non-ASCVD becomes dominant at more advanced stages of CKD [2, 3]. Atherosclerosis, beyond being a lipid disorder, is characterized by major inflammatory properties and has been implicated in the pathogenesis of arterial plaque formation and rupture and in clinical outcomes [4, 5]. Chronic inflammation, oxidative stress, and endothelial dysfunction separately and through their crosstalk have direct implications in the pathogenesis of atherosclerosis in CKD patients and have been established as nontraditional cardiovascular risk factors in this frail population [6–9].

Dyslipidemia, on the other hand, is one of the traditional cardiovascular risk factors. Lipid metabolism dysregulations are well recognized in patients with CKD [10, 11]. The common pattern of lipid disorders in a CKD patient consists of increased levels of triglycerides (TG) and lipoprotein (a) (Lp(a)), reduced levels of high-density lipoprotein cholesterol (HDL-C), and normal or slightly reduced total cholesterol (T-Chol) and low-density lipoprotein cholesterol (LDL-C) [10, 11]. Proprotein convertase subtilisin/kexin type 9 (PCSK9) has been identified as a central regulator of the LDL-receptor (LDL-R) expression by binding on the hepatocyte LDL-R, causing a reduction in the number of LDL-R and a subsequent enhancement in circulating LDL-C levels [12]. In CKD patients, existing literature is conflicting about possible association of PCSK9 plasma levels with the renal function. In the largest published observational study in two independent cohorts of nondialysis patients, Rogacev et al. showed no association of PCSK9 with renal function or with cardiovascular mortality [13]. Monoclonal antibodies against PCSK9 (PCSK9i) have emerged as safe and efficient hypolipidemic agents. In large clinical trials, PCSK9i manage to reduce LDL-C to target levels and Lp(a) levels by 20-30% with additional cardiovascular benefit when added to statin treatment in high-risk populations [14]. Nevertheless, efficacy of the PCSK9i alirocumab in CKD stage 3 patients was estimated in an analysis that pooled data from eight phase III ODYSSEY trials showing an efficacy and safety profile similar to that of patients with preserved eGFR [15].

Beyond the key role of PCSK9 on lipid disorders and management, emerging evidence points out its determining implication in the inflammatory arm of atherosclerosis. Experimental data have shown the upregulatory effect of proinflammatory and oxidative stress factors on the PCSK9 expression and vice versa, accordingly the possible modulatory role of PCSK9 on inflammation mediators [16]. Tumor necrosis factor alpha (TNF-*α*) resulted in the induction of PCSK9 mRNA and protein expression in HepG2 cells and vascular smooth muscle cells (VSMCs) [17], while oxidized low-density lipoprotein (ox-LDL) upregulated the PCSK9 expression in different cells, such as vascular endothelial cells (ECs), VSMCs, macrophages, and dendritic cells [18–23]. The administration of lipopolysaccharide in a PCSK9 knockout mice (PCSK9^−/−^) reduced plasma levels of TNF-*α* and interleukin 6 and 10 (IL-6, IL-10) [24], whereas knockdown of PCSK9 inhibited the inflammatory response in macrophages promoted by ox-LDL [19]. Moreover, PCSK9 has been implicated in endothelial dysfunction by upregulating the expression of intercellular adhesion molecule-1 (ICAM-1) and vascular cell adhesion molecule-1 (VCAM-1) in endothelial cells [16].

Experimental results are supported only by a limited number of clinical studies investigating the putative role of PCSK9 in the inflammatory process. Plasma levels of inflammatory cytokines, TNF-*α*, IL-6, and IL-8 were decreased in septic patients with a PCSK9 loss of function allele [24] while patients with bacteremia had increased PCSK9 plasma levels in a direct association with CRP [25]. Moreover, CRP as a marker of inflammation and ASCVD has been positively associated with plasma levels of PCSK9 both in patients with acute coronary syndromes and stable coronary disease [26]. In the same line, fibrinogen levels have been associated with PCSK9 levels in patients with stable coronary disease independently of traditional cardiovascular risk confounding [27].

Despite the emerging role of PCSK9 in ASCVD [28], there is lack of robust clinical data investigating the interplay of PCSK9 with markers of inflammation, oxidative stress, and endothelial dysfunction in high-risk patients such as CKD patients. Having this in mind, we designed a cross-sectional, observational study to investigate possible association of PCSK9 with inflammation markers, including high-sensitivity C-reactive protein (hs-CRP), IL-6, TNF-*α*, oxidative stress markers, 8-isoprostanes (8-epiPGF2a), and ox-LDL, and endothelial dysfunction markers such as (sICAM-1) and (sVCAM-1) in a nondialysis population of stable CKD patients. As a secondary outcome, we investigated the possible association of PCSK9 levels with renal function markers, lipid metabolism markers, and echocardiographic indices.

## 2. Patients and Methods

### 2.1. Participants

Ninety-two stable, CKD stage II-*Ι*V adults from the outpatient clinics of the Nephrology Department of our Tertiary Hospital were included in this single-center, cross-sectional, observational study. Twenty healthy volunteers attending the Outpatient Obesity and Lipid Clinic of the University Hospital of Ioannina, Greece, were recruited for the study in order to serve as a control group. The exclusion criteria were a recent major cardiovascular event (within the past 3 months before recruitment), active infection or history of infection in the last month, severe heart failure (NYHA IV) and/or severe valvulopathy, hepatic cirrhosis, active malignancy, and current immunosuppression treatment. All participants after being informed in detail provided signed informed consent to participate in the study. The study was approved by the Scientific Committee of the University Hospital of Ioannina, Greece, while all requirements of Helsinki Declaration were met.

### 2.2. Anthropometric Variables—Biochemical Parameters

On recruitment, all patients underwent a detailed review of their medical history and a careful clinical examination. The protocol of the study included recording demographic characteristics, smoking habits and alcohol consumption, primary renal disease, history of comorbidity and medication, anthropometric measurements, and measurement of blood pressure and heart rate. A routine full hematologic and biochemical screening was performed. All laboratory measurements were carried out after an overnight fast while water consumption was allowed. Plasma (using EDTA as an anticoagulant) and serum were prepared from blood samples and stored in aliquots at -80°C. Lipid profile assessment included serum levels of T-Chol, TC, and HDL-C that were determined enzymatically on an Olympus AU600 clinical chemistry analyzer (Olympus Diagnostica, Hamburg, Germany), LDL-C was calculated using the Friedewald formula, and Lp(a), apolipoprotein A1 (APO-A1), and apolipoprotein B (APO-B) were measured with a Behring Holding GmbH analyzer (Liederbach, Germany). All the above measurements were conducted in the laboratory facilities of the University Hospital of Ioannina. For the calculation of the estimated glomerular filtration rate (eGFR, ml/min/1.73 m^2^), the Chronic Kidney Disease Epidemiology Collaboration (CKD-EPI) equation was used [29]. Proteinuria was estimated in a 24-hour urine collection (UPR, mg/24 h).

### 2.3. Determination of PCSK9

Plasma PCSK9 concentration was determined by a quantitative sandwich enzyme immunosorbent assay using a commercially available kit according to the instructions provided by the manufacturer (R&D Systems, Inc.) as we previously described [30]. The intra-assay coefficient of variation ranges from 4.1 to 6.5, and the interassay coefficient of variation ranges from 4.1 to 6.0.

### 2.4. Determination of 8-epiPGF2a

Serum levels of 8-epiPGF2a were determined by means of a competitive ELISA using a commercially available kit (Cayman Chemicals, Ann Arbor, MI USA), as we previously described [31, 32]. This method has a specificity of 100% for 8-epiPGF2a, while having minimal crossreactivity with other compounds, mostly 8-isoPGF3a.

### 2.5. Determination of ox-LDL

Plasma levels of ox-LDL were measured by a competitive enzyme-linked immunosorbent assay using a specific murine monoclonal antibody (4E6) according to the instructions provided by the manufacturer (Mercodia, Uppsala, Sweden) as we previously described [32]. Intra- and interassay coefficients of variation were 6.0% and 7.0%, respectively.

### 2.6. Determination of IL-6 and TNF-*α*

Serum IL-6 and TNF-*α* were measured by a high-sensitivity ELISA (Quantikine HS human IL-6 and TNF-*α*, Research & Diagnostic Systems Europe Ltd., Abington UK). The sensitivity of the ELISA system was less than 0.5 pg/ml for both IL-6 and TNF-*α*.

### 2.7. Determination of hs-CRP, sICAM-1, and sVCAM-1

Serum hs-CRP levels were measured by high-sensitivity immunoturbidimetry (Cobas Integra 800, Roche). Serum levels of the molecules sICAM-1 and sVCAM-1 were measured by a sandwich enzyme immunoassay technique (ELISA) using commercially available standard kits (Quantikine human sICAM-1 and VCAM-1, Research & Diagnostic Systems Europe Ltd., Abington UK). The sensitivity of the ELISA system was less than 2 ng/ml for both sICAM-1 and sVCAM-1.

### 2.8. Echocardiography

Left ventricular mass (LVM) was assessed by 2D-mode echocardiogram usually within one week and no longer than one month from study entry, by a single cardiologist who followed a predefined protocol for the recordings and measurements and was blinded to the clinical and biochemical data. LV mass was estimated with Devereux formula, and LV mass index (LVMI) was calculated by dividing LV mass with patient's BMI [LVMI = LV mass (g)/BSA (m^2^)] [33, 34]. Left ventricle ejection fraction (LVEF) and left ventricle shortening fraction (LVFS) were estimated as well.

## 3. Statistical Analysis

Frequencies and percentages were used to describe all categorical data collected, while means with standard deviation (normally distributed data) and medians with interquartile range (IQ, nonnormally distributed data) for the scale measurements. Pearson's correlation coefficient or Spearman's rho, depending on the data distribution, was used to assess linear relationships, while differences in the PCSK9 levels between dichotomous data were examined with the independent samples *t*-test, after checking the normality assumption under the Kolmogorov–Smirnov test. Multiple regression analysis was carried out to test the effect of all independent parameters for their effect on plasma PCSK9 levels. Included parameters were all with a *p* < 0.2 in the univariate analysis (sex, diabetes mellitus, statin treatment, logUPR, Lp(a), TG, CRP, fibrinogen, ox-LDL, and sICAM-1). The level of significance was set at 0.05, and the analysis was conducted using the SPSS v23.0 software.

## 4. Results

Patients' mean age was 66 years, and 51 (55.4%) were males. Demographic, clinical, and laboratory data and comorbidities of the 92 patients are shown in Table 1. The mean eGFR was 47.3 ± 25.7 mg/ml/1.73 m^2^, and the median proteinuria was 323 mg/24 h (IQR, 140-1148 mg/24 h).

The mean PCSK9 plasma level in CKD patients was 278.10 ng/ml in comparison with significantly lower PCSK9 level in 20 control subjects (mean value 156 ± 43 ng/ml) (Table 2). Median hs-CRP was 1.0 (0.3-4.3) mg/l, mean fibrinogen was 433 mg/dl, median IL-6 was 3.1 (1.9-4.5) pg/ml, median TNF-*α* was 1.9 (1.4-3.0) pg/ml, mean 8-epiPGF2a was 110 pg/ml, ox-LDL was 79.1 U/l, median sICAM-1 was 240 (200-317) ng/ml, and mean VCAM-1 was 917.3 ng/ml.

### 4.1. Associations of Kidney Function Parameters

Age, systolic BP, UPR, uric acid, PTH, and LVMI increased with decreasing eGFR (*p* < 0.05 for age and *p* < 0.001 for all other parameters), whereas BMI and hemoglobin decreased with advanced CKD (*p* < 0.001 for both). Among lipid profile markers, only APO-A1 was significantly associated with eGFR in a direct fashion (*p* = 0.001). Kidney function expressed as eGFR showed significant inverse association with fibrinogen (*p* < 0.001), IL-6 (*p* < 0.05), TNF-*α* (*p* < 0.001), 8-epiPGF2a (*p* < 0.001), and sVCAM-1 (*p* < 0.001).

Urine protein daily excretion was strongly associated (in a direct fashion) with TG (*p* < 0.001), LVMI (*p* < 0.001), 8-epiPGF2a (*p* = 0.007), and sVCAM-1 (*p* < 0.001).

We did not find a significant association between PCSK9 levels and markers of the kidney function in the nondialysis CKD population of our study.

### 4.2. Associations between Inflammation, Oxidative Stress, and Endothelial Dysfunction Markers

Associations between inflammation, oxidative stress, and endothelial dysfunction markers are shown in Table 3. High-sensitivity CRP was found to have a direct association with fibrinogen (*p* < 0.001), IL-6 (*p* < 0.001), 8-epiPGF2a (*p* < 0.001), and sICAM-1 (*p* < 0.001). Interleukin-6 significantly associated in a direct manner with fibrinogen (*p* < 0.001), TNF-*α* (*p* = 0.001), and sVCAM-1 (*p* < 0.001), while TNF-*α* and sVCAM-1 had a direct strong association (*p* < 0.001). sVCAM-1 was found to correlate directly with sICAM-1 as well (*p* < 0.001). Significant associations of oxidative stress markers were direct association of 8-epiPGF2a with hs-CRP, sICAM-1, and sVCAM-1 (*p* < 0.001, *p* = 0.001, and *p* = 0.003, respectively), while ox-LDL associated directly with lipid metabolism parameters, TG, T-Chol, LDL-C, and APO-B (*p* < 0.001 for all) and with Lp(a) (*p* = 0.02).

### 4.3. Associations of PCSK9 with Lipid Metabolism Parameters, Inflammation, Oxidative Stress, and Endothelial Dysfunction Markers

**Figure 1:**
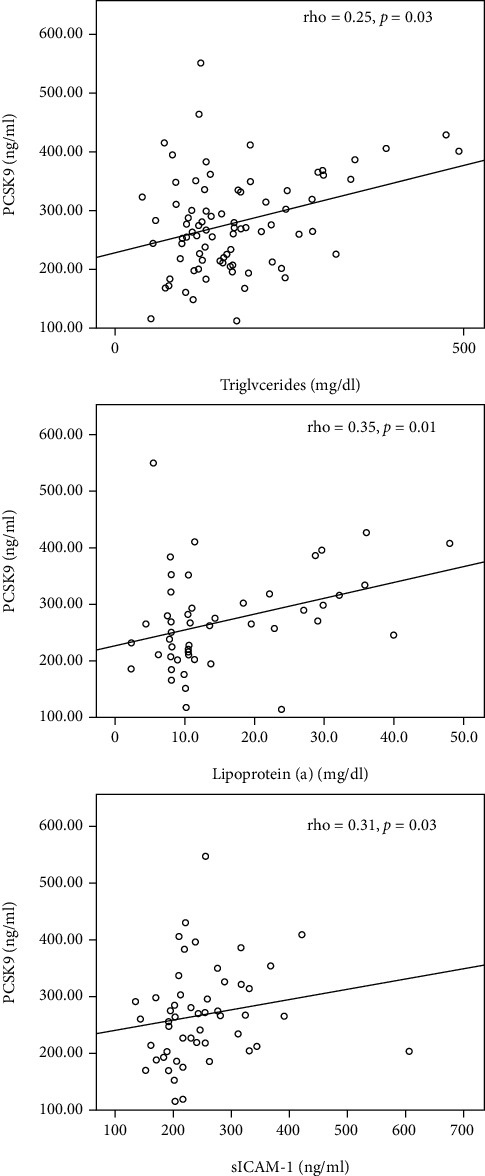
Significant associations of PCSK9 with triglycerides, Lp(a), and sICAM-1 in CKD patients.

PCSK9 levels were directly associated with TG (*p* = 0.03), Lp(a) (*p* = 0.01), and ICAM-1 (*p* = 0.03) (Figure 1). There was a significant positive correlation between PCSK9 levels and statin treatment. Patients on statin treatment had higher PCSK9 levels in comparison with those who did not (318.19 ng/ml vs. 253.57 ng/ml, *p* < 0.001). In our studied CKD population of stages II-IV, PCSK9 plasma levels were not associated with kidney function parameters, diabetes mellitus, and cardiovascular comorbidity or with echocardiography indices.

Multiple regression analysis, as described above, was conducted to test all independent parameters for their effect on plasma PCSK9 levels. The results showed a statistically significant effect of the Lp(a) values, as well as of statin intake. Specifically, for each unit of higher Lp(a), an increase by 3.082 is expected for the PCSK9 values (95% CI: 0.935-5.228, *p* = 0.006). At the same time, patients receiving statins are expected to have on average 63.8 ng/ml higher PCSK9 values compared to patients not receiving statins (95% CI: 14.6-113.5, *p* = 0.012).

## 5. Discussion

This study shows that in nondialysis CKD patients, plasma levels of PCSK9 are directly associated with the endothelial dysfunction biomarker sICAM-1 and with Lp(a), an established risk factor for myocardial infraction and cardiovascular death in CKD patients [35]. A plethora of experimental and clinical studies have established the central role of PCSK9 in lipid metabolism while robust evidence supports the implication of PCSK9 in the inflammatory nature of atherosclerosis [16].

Inflammation, oxidative stress, and endothelial dysfunction hold a key role in ASCVD in CKD patients. Studies investigating the role of PCSK9 in atherosclerosis beyond LDL-C regulation completely lack in the CKD high-risk population. In this clinical study, for the first time, we tested the possible association of PCSK9 levels with inflammation, oxidative stress, and endothelial dysfunction biomarkers in a cohort of CKD nondialysis patients. We found that sICAM-1 levels were directly associated with PCSK9 levels. The increased levels of sICAM-1 are shown to be an independent predictor of mortality in predialysis patients with cardiovascular disease [36, 37]. In the basic step of atherosclerotic vascular damage, LDL-C stimulates endothelial cells in the inner layer which express on their surface ICAM-1 and VCAM-1 and further promote the adhesion of circulating inflammatory leucocytes. Serum PCSK9 levels have been evaluated in healthy participants and showed an independent association with arterial stiffness, a well-established risk factor for atherosclerotic cardiovascular disease [38]. Moreover, in patients undergoing coronary angiography for acute coronary syndrome or stable angina, PCSK9 was found to linearly associate with the fraction and amount of necrotic core tissue in coronary atherosclerosis, independently of serum LDL cholesterol levels and statin use [39]. In an animal model, administration of lipopolysaccharides in PCSK9 knockout mice diminished the expression of VCAM-1 from endothelial vascular cells compared with wild type [16]. Release of ICAM-1 from vascular endothelial cells was reduced with PCSK9i alirocumab and anti-PCSK9 vaccine AT04 in the APOE∗3Leiden.CETP transgenic mouse model for hyperlipidemia and atherosclerosis [40, 41].

Among studies estimating PCSK9 levels in nondialysis CKD patients, only two reported the association of a marker of inflammation hs-CRP with PCSK9 levels in this population. In the first one, with 44 CKD patients (eGFR 20.2 ml/min/1.73 m^2^), the authors reported a rather weak direct correlation between the two markers (*r* = 0.26, *p* < 0.05) [42]. In the largest one, Rogacev et al. assessed PCSK9 levels in two cohorts of CKD patients (CARE FOR HOMe cohort and LURIC cohort) and found no correlation between PCSK9 and hs-CRP levels nor with eGFR [13]. In accordance with Rogacev et al., we did not find any correlation between plasma PCSK9 levels and neither hs-CRP nor with kidney function markers, eGFR and UPR, in our patients. Most of the available studies showed no correlation between PCSK9 and eGFR. Interestingly, one small crossover study has shown that plasma PCSK9 can be manipulated in response to therapeutic interventions, which have other hemodynamic benefits such as endothelin antagonism [43]. Moreover, a recent experimental study in a LDL-R+/- mouse model investigated the potential effect of a vaccine targeting PCSK9 (PCSK9Q*β*-003) on hypercholesterolemia and kidney fibrosis. According to their results, vaccination with PCSK9Q*β*-003 had a positive effect on lipid accumulation and renal fibrosis through regulation of fatty acid *β*-oxidation [44]. PCSK9 is suggested to be involved in the dyslipidemia and proteinuria of nephrotic syndrome in CKD [45, 46]. The results of a very recent study of Molina-Jijon et al. showed that the kidney PCSK9 expression was enhanced in the collecting duct of nephrotic patients and animals, supporting the hypothesis that the kidney could be a major source for plasma PCSK9 in nephrotic syndrome. In our study, the vast majority of our patients were not nephrotic [47].

We found that among lipid metabolism parameters, plasma PCSK9 in our CKD patients correlated directly with TG and Lp(a) concentrations. Associations of PCSK9 with lipid profile markers are not consistent in published studies in the different high cardiovascular risk populations [11–13]. Dyslipidemia in CKD is characterized mainly by high TG levels and Lp(a), reduced levels of HDL-C, and normal or slightly reduced T-Chol and LDL-C [10, 11]. Pathogenetically, PCSK9 enhances the degradation of hepatic LDL-R, resulting in an increase in LDL cholesterol levels. We have found no association between plasma PCSK9 levels and T-Chol, HDL-C, or LDL-C in our patients. Rogacev et al. did not find either, while we both found a direct correlation with TG levels [13]. The absence of an association between PCSK9 and LDL-R might suggest that only a segment of circulating PCSK9 can mediate the degradation of LDL-R in addition to the fact that 36% of our patients were receiving statins. In our study, patients receiving statin had on average 63.8 ng/ml higher PCSK9 values compared to patients not receiving statins. It is known that statins inhibit 3-hydroxy-3-methylglutaryl coenzyme A reductase (HMGCoAR), a rate-limiting enzyme in cholesterol biosynthesis, and have been shown to significantly increase PCSK9 mRNA in HepG2 cells and primary human hepatocytes through activation of the sterol regulatory element-binding protein-2 (SREBP-2) pathway [48]. Increased levels of PCSK9 levels in patients on statin treatment could indicate a possible implication of PCSK9 in the lipoprotein TG contents by lipoprotein lipase regulation in CKD patients and an effect on high cardiovascular burden in this patient population. Nevertheless, more dedicated studies are required in order to elucidate the role of PCSK9 along with the role of PCSK9i in CKD.

Lipoprotein (a), a subtype of LDL-C, is an established risk factor for ASCVD in the general and CKD population [11, 49]. The metabolic pathways of Lp(a) production and clearance are not completely elucidated yet. Nevertheless, robust clinical evidence showed that PCSK9i reduced levels of Lp(a) by 20-30% and contributed to reduction of incident major cardiovascular event. Proposed mechanisms are either by increasing catabolism or by reducing production [49, 50]. In accordance to our results, a direct association between PCSK9 levels and Lp(a) was found by Bermudez-Lopez et al. in a cross-sectional study including 209 nondiabetic CKD patients not receiving statin treatment [51].

In this study, in line with our previous publications, we found significant associations between severity of CKD and inflammation (IL-6, TNF-*α*), oxidative stress (8-epiPGF2), and endothelial dysfunction (fibrinogen, sICAM-1, and sVCAM-1) markers and interesting correlations among these novel biomarkers as part of their well-recognized interplay in the uremic milieu [52, 53]. The results of this study did not demonstrate any association between PCSK9 with the majority of these biomarkers. On the other hand, a growing body of experimental evidence, as aforementioned, highlights the key role of PCSK9 in the pathogenesis of atherosclerosis by its implication in inflammation, apoptosis, oxidative stress, and endothelial damage [16, 54]. Clinical observational data are conflicting regarding the role of PCSK9 as a predictive risk factor for mortality in CKD patients. In a recent prospective study, Strålberg et al. included 265 patients starting dialysis and found a U shape association of PCSK9 levels with all-cause mortality independently of a number of confounders [55]. In contrast to this study, Rogacev et al. failed to demonstrate PCSK9 as a prognostic risk factor for cardiovascular outcomes in nondialysis CKD patients [13].

Our study has some strengths and limitations. To our knowledge, this is the only clinical observational study examining the possible association between PCSK9 with a panel of inflammation, oxidative stress, and endothelial dysfunction markers in nondialysis CKD patients. The main limitations of our study are rather small sample size and cross-sectional observational study design. Again, due to the observational nature of our findings, causality cannot be inferred from our data.

## 6. Conclusion

The emerging experimental data indicate that PCSK9 might have additional roles other than regulating blood LDL-C, while PCSK9i have emerged as a very promising category of hypolipidemic agents for the treatment of high-risk populations unable to achieve LDL-C target levels. Clinical data in CKD patients are scarce and not consistent regarding the role of PCSK9 in the inflammatory arm of atherosclerosis and on cardiovascular outcome. In our study, we showed that PCSK9 is possible to be a piece of the complex, unravel puzzle of ASCVD in CKD. As CKD patients remain a population with unmet needs in the management of dyslipidemia and cardiovascular morbidity and mortality, PCSK9 might be an interesting therapeutic target for the treatment of atherosclerotic disease beyond LDL-C regulation. Further prospective research is warranted to elucidate the effects of PCSK9 and PCSK9i in patients with reduced renal function.

## Figures and Tables

**Table 1:** Demographic characteristics, clinical and laboratory data, and comorbidities of the 92 CKD patients and the 20 controls.

Parameters	CKD patients (*n* = 92)	Controls [20]
Age (years)	65.8 ± 12.45	35 ± 6
Gender (male), *n* %	51 (55.4%)	6 (30%)
BMI (kg/m^2^)	47.29 ± 25.68	22.7 ± 2.0
SBP (mmHg)	142 ± 19	111 ± 12
DPB (mmHg)	81 ± 11	67 ± 8
Hypertension, *n* %	80 (74%)	0
Diabetes mellitus, *n* %	26 (28.3%)	0
History of CAD, *n* %	25 (27%)	0
Statin treatment, *n* %	33 (36%)	0
eGFR/CKD-EPI (ml/min/1.73 m^2^)	47.3 ± 25.7	89.5 ± 18.3
UPR (mg/24 h)	323 (140, 1148)	—
T-Chol (mg/dl)	209 ± 47	172 ± 27
TG (mg/dl)	163 ± 88	67 ± 32
HDL (mg/dl)	52 ± 15	57 ± 11
LDL (mg/dl)	124 ± 39	102 ± 21
Lp(a) (mg/dl)	11.1 (8.0, 29.3)	8 (1-24)
APO-A1 (mg/dl)	139 ± 30	159 ± 23
APO-B (mg/dl)	93 ± 28	65 ± 15
Albumin (mg/dl)	4.23 ± 0.40	4.4 ± 0.30
Uric acid (mg/dl)	6.82 ± 1.64	5.1 ± 1.35
PTH pg/ml	74 (47, 121)	—
Hb (g/dl)	13.0 ± 1.6	13.6 ± 1.4
HbA1c (%)	6.1 (5.7, 7.2)	—
LVMI (g/m^2^)	135.7 ± 47.1	—
EF (%)	69 ± 10	—
FS (%)	37 ± 8	—

**Table 2:** Levels of PCSK9, inflammation, oxidative stress, and endothelial dysfunction markers in the 92 CKD patients and the 20 controls.

PCSK9 (ng/ml)	278.10 ± 80.2	156.2 ± 43.1
CRP (mg/l)	1.0 (0.3, 4.3)	1.0 (1.0, 2.2)
Fibrinogen (mg/dl)	433 ± 174	312 ± 136
IL-6 (pg/ml)	3.1 (1.9, 4.5)	0.8 (0.5, 2.6)
TNF-*α* (pg/ml)	1.9 (1.4, 3.0)	0.7 (0.1, 3.3)
8-epiPGF2a (pg/ml)	110 (92, 138)	45 ± 19
ox-LDL (U/l)	79.1 ± 23.9	42 ± 15
sICAM-1 (ng/ml)	240 (200, 317)	224 ± 20
sVCAM-1 (ng/ml)	917.3 ± 377.1	602.9 ± 145

**Table 3:** Associations between inflammation, oxidative stress, endothelial dysfunction, and lipid metabolism markers in the CKD patients.

		Spearman rho	*p* value
hs-CRP	Fibrinogen	0.44	<0.001
	8-epiPGF2a	0.32	<0.001
	IL-6	0.27	<0.001
	sICAM-1	0.35	<0.001
IL-6	Fibrinogen	0.32	<0.001
	TNF-*α*	0.25	0.001
	sVCAM-1	0.31	<0.001
TNF-*α*	sVCAM-1	0.35	<0.001
sVCAM-1	sICAM-1	0.27	<0.001
8-epiPGF2a	sICAM-1	0.26	0.001
	sVCAM-1	0.23	0.003
ox-LDL	TG	0.30	<0.001
	T-Chol	0.68	<0.001
	LDL-C	0.71	<0.001
	APO-B	0.64	<0.001
	Lp(a)	0.20	0.02

## Data Availability

The demographic, clinical, and laboratory data of the patients used to support the findings of this study are available from the corresponding author upon request. In any case, personal data protection will be ensured.
